# Prediction of Chronic Stress and Protective Factors in Adults: Development of an Interpretable Prediction Model Based on XGBoost and SHAP Using National Cross-sectional DEGS1 Data

**DOI:** 10.2196/41868

**Published:** 2023-05-16

**Authors:** Arezoo Bozorgmehr, Birgitta Weltermann

**Affiliations:** 1 Institute of General Practice and Family Medicine University Hospital Bonn University of Bonn Bonn Germany

**Keywords:** artificial intelligence, machine learning, prognostic, model, chronic stress, resilience factors, interpretable model, explainability, stress, disease, diabetes, cancer, dataset, clinical, data, gender, social support, support, intervention, SHAP

## Abstract

**Background:**

Chronic stress is highly prevalent in the German population. It has known adverse effects on mental health, such as burnout and depression. Known long-term effects of chronic stress are cardiovascular disease, diabetes, and cancer.

**Objective:**

This study aims to derive an interpretable multiclass machine learning model for predicting chronic stress levels and factors protecting against chronic stress based on representative nationwide data from the German Health Interview and Examination Survey for Adults, which is part of the national health monitoring program.

**Methods:**

A data set from the German Health Interview and Examination Survey for Adults study including demographic, clinical, and laboratory data from 5801 participants was analyzed. A multiclass eXtreme Gradient Boosting (XGBoost) model was constructed to classify participants into 3 categories including low, middle, and high chronic stress levels. The model’s performance was evaluated using the area under the receiver operating characteristic curve, precision, recall, specificity, and the *F*_1_-score. Additionally, SHapley Additive exPlanations was used to interpret the prediction XGBoost model and to identify factors protecting against chronic stress.

**Results:**

The multiclass XGBoost model exhibited the macroaverage scores, with an area under the receiver operating characteristic curve of 81%, precision of 63%, recall of 52%, specificity of 78%, and *F*_1_-score of 54%. The most important features for low-level chronic stress were male gender, very good general health, high satisfaction with living space, and strong social support.

**Conclusions:**

This study presents a multiclass interpretable prediction model for chronic stress in adults in Germany. The explainable artificial intelligence technique SHapley Additive exPlanations identified relevant protective factors for chronic stress, which need to be considered when developing interventions to reduce chronic stress.

## Introduction

Chronic stress has many negative effects, primarily on mental health, for example burnout and depression [1]. Long-term chronic stress is associated with various illnesses including cardiovascular disease, diabetes, cancer, and asthma [2-5]. High chronic stress is prevalent with multiple mental health problems in the German population, and this value has increased to 61.1% [6]. However, the vast majority of the population does not develop high chronic stress. While most research has focused on the development of pathology and risk factors, it is paramount to better understand protective factors that prevent chronic stress. In our prior study [7] with 764 participants including general practitioners (GPs) and practice assistants (PrAs) from 136 German general practices, we analyzed the level of strain due to stress stratified for personal, practice, and regional characteristics. We showed that GPs and PrAs, who individually applied more than 5 measures regularly to compensate for stress, had markedly lower stress levels as measured by the Screening Scale of the Trier Inventory for the Assessment of Chronic Stress (TICS-SSCS) instrument [8].

The psychological construct of resilience, developed over the last decades, addresses this perspective. The American Psychological Association (in 2014) defines resilience as “the process of adapting well in the face of adversity, trauma, tragedy, threats or even significant sources of stress” [9]. Resilience in the context of chronic stress has been characterized by the ability to “bounce back from negative emotional experiences and by flexible adaptation to the changing demands of stressful experiences” [10]. It involves the ability to maintain healthy functioning in different domains of life, such as work and family. Holz et al [11] provided an overview of the current literature investigating the neural mechanisms of resilience focusing on social background. They discussed possible prevention and early intervention approaches targeting the individual and the social environment to lower the risk of psychiatric disorders and to foster resilience [11]. Schetter et al [12] reviewed the traditions of research and definitions of resilience to chronic stress in adults and gained an understanding of resilience in general. They developed a taxonomy of resilience resources to guide future research [12]. Other studies focused on neurobiological cascades involving, for example, enkephalins and associated opioid receptors, μ‑opioid peptide receptor, and δ‑opioid peptide receptor, to better understand the biological mechanisms of natural adaptation. Prospectively, this bares the potential for effective preventive or therapeutic strategies [13].

To better understand the chronic stress in epidemiological studies, machine learning (ML) offers new approaches to evaluate and model complex relationships in data [14,15]. ML strategies are based on algorithms, which describe the relationships between variables. Two areas in medicine that benefit from ML techniques are diagnosis and outcome prediction [16,17]. Focusing on chronic stress prediction, our prior study [18] compared 4 supervised ML classifiers and 1 standard approach based on data of 550 PrAs from 136 German general practices. We showed that all 4 ML approaches, especially random forest, provided more accurate models for predicting chronic stress than standard regression analysis [18].

Aiming at an interpretable multiclass ML model for predicting chronic stress, we developed an eXtreme Gradient Boosting (XGBoost) model based on nationally representative German Health Interview and Examination Survey for Adults (DEGS1) data. The unified framework SHAP (SHapley Additive exPlanations) is used to interpret the prediction model and to identify factors protecting against chronic stress.

## Methods

### Overview

This study used nationally representative data from the DEGS1 study, which is a part of the health monitoring program of the Robert Koch Institute, Berlin, Germany. It was conducted from 2008 to 2011 by means of interviews, examinations, and tests among the German population aged 18-79 years (n=8151). The DEGS1 data set, which is available for public use on request, included measurements for chronic stress among 5801 respondents aged 18 to 64 years [6,19].

### Primary Outcome

Chronic stress was assessed using the 12-item German short version of TICS-SSCS (n=5850) [6]. It was developed by Schultz et al [8] based on the systemic-requirement-resource model of health [8,20]. The 12-item scale addresses 5 stress areas: chronic worrying, work overload, social overload, excessive demands of work, and lack of social recognition. Its internal consistency showed a Cronbach α of .91 and a good to very good reliability with values ranging from .84 to .91 (mean α=.87) [8]. All 12 questionnaire items use a 5-point Likert scale answer format (0=“never” to 4=“very often”) to measure chronic stress in the past 3 months [21,22]. A sum score (scale 0-48) was calculated for each participant, which is categorized in 3 classes based on a reference population with the TICS-SSCS: 1-11 (≤median)=low stress, 12-22=middle stress, and >22=high stress (≥90th percentile). This multiclass outcome is the recommended DEGS1 approach [6].

### Predictors

In addition, the DEGS1 data set included variables on sociodemographic characteristics, chronic diseases (eg, coronary heart disease, stroke, diabetes mellitus, depression, and anxiety disorder), living conditions, health-related behavior, preventive measures, and general health. Based on a literature review and using the Powershap feature selection method, 34 features were included in this analysis. Table 1 depicts descriptive information about the variables used.

**Table 1:** Demographic, clinical, and workplace characteristics of the German Health Interview and Examination Survey for Adults study participants (N=5801).

Demographic characteristics				Values
**Continuous variables, mean (SD; range)**				
	Age (years)		42 (13.11; 18-64)	
	Number of persons in the household		3 (1.34; 1-11)	
	Sleep hours per night in the past 4 weeks		7 (1.19; 2-12)	
	Number of hospital nights in the past 12 months		1 (5.30; 0-150)	
	Number of sick days in the past 12 months		13 (38.01; 0-365)	
**Categorical variables**				
	Gender (female), n (%)		3081 (49.6)	
	**Marital status, n (%)**			
		Married living with partner or separately from partner	3697 (59.5)	
		Single	1957 (31.5)	
		Divorced	376 (6.1)	
		Widowed	136 (2.2)	
	Provides care to someone in need or seriously ill, n (%)		379 (6.1)	
	**Renting or living in own apartment/house, n (%)**			
		Rented apartment or house	2689 (43.3)	
		Own apartment or house	3268 (52.6)	
	**Satisfaction with living space, n (%)**			
		Very satisfied or satisfied	5269 (84.8)	
		Neither satisfied nor dissatisfied	608 (9.8)	
		Dissatisfied or very dissatisfied	295 (4.8)	
	**Residential area satisfaction, n (%)**			
		Very satisfied or satisfied	5091 (81.9)	
		Neither satisfied nor dissatisfied	727 (11.7)	
		Dissatisfied or very dissatisfied	320 (5.2)	
	**General state of health, n (%)**			
		Very good or good	4942 (79.5)	
		Average	1134 (18.3)	
		Poor or very poor	116 (1.8)	
	**Intake of sleeping pills in the past 4 weeks, n (%)**			
		Never	5919 (95.3)	
		Less than 1 time	100 (1.6)	
		1 time or 2 times	73 (1.2)	
		3 times or more	86 (1.4)	
	**Social support, n (%)**			
		Low support	653 (10.5)	
		Average support	3082 (49.6)	
		Strong support	2451 (39.5)	
	**Health behavior consultation in the past 12 months, n (%)**		1873 (30.4)	
		Has general practitioner	5497 (88.5)	
		Visited to general practitioner in the past 12 months	4870 (78.4)	
		Visited to neurologist in the past 12 months	463 (7.5)	
	**Frequency of alcohol consumption, n (%)**			
		Never	744 (12.0)	
		1 time per month or less	1186 (19.1)	
		2-4 times per month	1998 (32.2)	
		2-3 times per week	1453 (23.4)	
		4 times per week or more	811 (13.1)	
	**Tobacco use, n (%)**			
		Yes, daily	1701 (27.4)	
		Yes, occasionally	433 (7)	
		Not anymore	1664 (26.8)	
		Never smoked	2400 (38.7)	
	**Comorbidities, n (%)**			
		Has hypertension	1625 (26.2)	
		Has diabetes	271 (4.4)	
		Has migraine	712 (11.5)	
		Has depression	682 (11)	
		Has anxiety disorder	327 (5.3)	
		Has burnout syndrome	292 (4.7)	
		Has one or more long-term chronic diseases	1418 (22.8)	
	**Prevention programs or sport activities, n (%)**			
		Participated in prevention program in the past 12 months	988 (15.9)	
		Participated in relaxation or stress management program	188 (3)	
		Participated in gymnastics, fitness, or balance sports program	832 (13.4)	
		Participated in alcohol cessation program	7 (0.1)	
		Participated in smoking cessation program	17 (0.3)	
		Participated in weight reduction or a healthy diet program	167 (2.7)	
	**Sports activities per week (in the past 3 months), n (%)**			
		No sports activity	1954 (31.5)	
		Up to 2 hours per week	2584 (41.6)	
		Regularly, 2-4 hours per week	990 (15.9)	
		Regularly, more than 4 hours per week	645 (10.4)	

### Data Preprocessing

#### Data Normalization

The DEGS1 study features include both discrete and continuous values. When these features are combined, the range of the values differs. Therefore, the training data set was normalized using the min-max normalization method. This normalization technique accurately preserves all relationships in the data, thereby avoiding the introduction of bias [23].

#### Handling of Missing Data

For single features, missing values were low (<2%), yielding an overall missing rate of 13.91% in our data set. We used the K-Nearest Neighbors (KNN) approach to impute the missing variables. This method identifies the KNNs on the Euclidean distance. Missing values were replaced using a majority vote for discrete variables and weighted means for continuous features. All features are imputed simultaneously without the need to treat features individually [24].

#### Addressing the Imbalanced Data Set

For chronic stress, the distribution of classes was unequal (class 0: 52%, class 1: 38%, and class 2: 11%). This imbalanced multiclass classification was addressed using the Synthetic Minority Oversampling TEchnique to increase the frequency of near-miss data points within the training data set. This oversampling method randomly generated new instances of minority class to balance the number of classes without any additional information to the model [25].

#### Feature Selection

We used Powershap as a wrapper-based Shaply feature selection method. This technique is based on the core assumption that an informative feature will have a larger impact on the prediction compared to a known random feature [26].

### Machine Learning Approach: XGBoost

#### Overview

To predict chronic stress levels and detect factors protecting against chronic stress, we applied the decision tree–based ensemble ML technique, XGBoost [27,28]. XGBoost is a scalable and accurate implementation gradient boosting machine developed by the Distributed Machine Learning Community in the form of open-source libraries. It combines a recursive gradient boosting method called Newton boosting. Based on a decision tree model, it efficiently provides accurate predictions because each tree is boosted recursively and in parallel.

The ML technique generally aims to identify a relationship between the input ***X***={***x*_1_**, ***x*_2_**, … ***x*_n_**} and the output **Y**. For a given data set with **n** samples and **m** features, **K** additive functions are used in the XGBoost model to predict the output through the following estimation (equation 1) [27]:



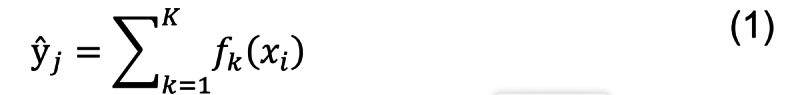



where **f_k_** ϵ {**f**(**x**) = **ω_q_**} (**q**: R**^m^** → **T**, **ω** **ϵ** R**^T^**) is the regression tree’s space, and **q** denotes the independent structure of each tree with **T** leaves. Each **f_k_** corresponds to an independent tree structure **q** and leaf weights **ω.** The following regularized objective is minimized to learn the set of functions (equation 2).



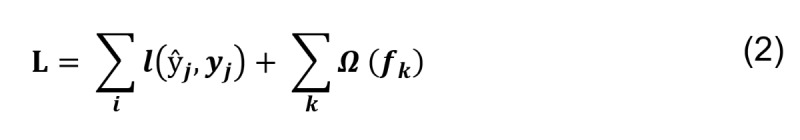



where **Ω** (**f**) = **γ** **T** + **½ λ** ||**ω**||**^2^**, **I** represents the model loss function, and **Ω** denotes the regularized term.

#### Hyperparameter Tuning

In this study, a grid-search approach from scikit-learn class “GridSearchCV” was applied toward the optimal tuning of XGBoost hyperparameters. The number of estimators was set to 1000 to represent the maximum number of trees created during the training phase. The Softmax function is used to convert logits of the XGBoost classifier into a probability distribution. Each element of the output lies in the interval (0,1) and the output elements sum up to 1. Table 2 summarizes the hyperparameters´ values used to the XGBoost model (see Multimedia Appendix 1).

**Table 2:** Main hyperparameters for the Extreme Gradient Boosting model.

Hyperparameter	Value
learning rate	0.3
Estimators, n	1000
max_depth	5
Subsample	0.8
min_child_weight	3
L2 regularization term (Lambda)	2
colsample-bytree	0.7
Objective	multi:softmax

#### K-Fold Cross-Validation

After preprocessing, the 34 features were fed into ML classifiers to train the model for classification. The data set was split into a “training” and a “validation” data set. We used the repeated K-fold cross-validation approach, repeating the mean performance across all folds and all repeats to reduce the bias in the model's estimated performance with K=10. K=10 was chosen as the optimal number of folds, which optimizes the time to complete the test while minimizing the bias and variance associated with the validation process.

### Model Performance Evaluation

To evaluate the method proposed in this study, we used the following most promising multiclass evaluation metrics: the area under the receiver operating characteristic curve (AUC), precision, recall, and *F*_1_-score. Multiclass classification works on data sets in which all classes are mutually exclusive. In a multiclass classifier, the evaluation measures of individual classes are averaged out to determine the performance on overall system across the data. We applied the macroaverage approach [29].

The receiver operating characteristic (ROC) curve was used to evaluate the performance of the classifier. For different classification thresholds, the macro true-positive rate (equation 3) is plotted against the macro false-positive rate (equation 4). The AUC indicates the classifier’s ability to distinguish between classes. The value of the AUC is in the range (0,1), in which 1 is for a perfect classifier. In this study, the ROC curve is plotted for each class broken down into a series of binary problems using the One-vs-Rest approach. The macroaverage is computed by summing the individual values for true positive, true negative, false positive, and false negative. Then, macroaverage scores of true positive instances (precision; equation 5), true positive rate (recall; equation 6), true negative rate (specificity; equation 7), and the harmonic mean of the precision and recall computed on each class (*F*_1_-score; equation 8) were computed. Mathematically, they are defined as follows:



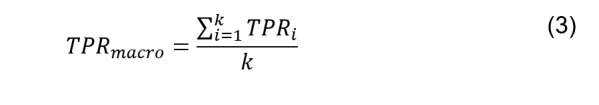





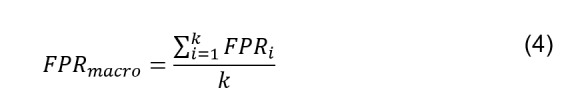





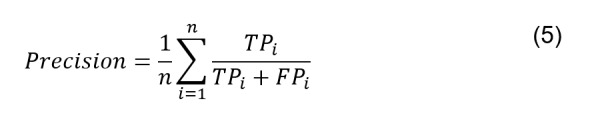





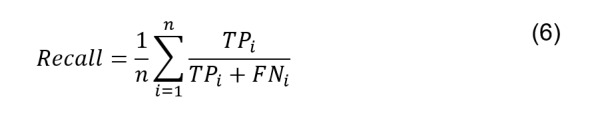





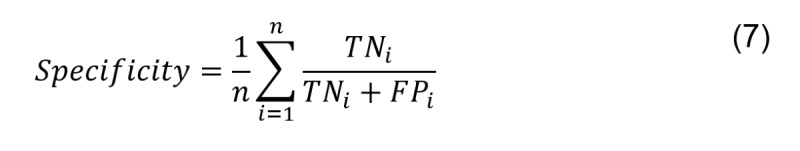





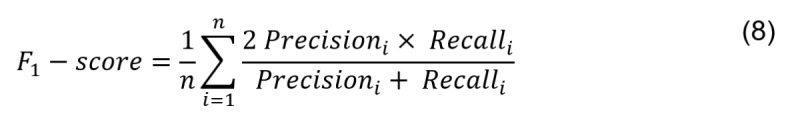



We used Python 3.7 (Python Software Foundation) to implement our ML framework. In addition, several libraries from the python data science ecosystem were used to execute the experiments and the integrated development environment PyCharm. To implement the Powershap feature selection method, we used the Powershap Python library. The scikit-learn package (version 1.0.2) was used to train and evaluate the ML classifier. SHAP tool (version 0.40.0) was used to assess the explainability the model; that is, to identify factors protecting against chronic stress.

In addition to the performance evaluation, this study maximizes the interpretability of the underlying models. It focuses particularly on the explainability of the model, which can serve as an indispensable tool in the era of precision medicine.

### Model Interpretation: SHAP

Per our understanding, the interpretation of the prediction models is as crucial as the prediction accuracy because it extracts information that significantly affects outcomes and identifies the factors protecting against chronic stress from subjects with lower chronic stress. However, the ensemble learning method XGBoost represents a black-box model. To overcome this problem, Lundberg [30,31] proposes the SHAP approach for interpreting predictions of complex models created by different techniques; for example, NGBoost, CatBoost, XGBoost, LightGBM, and scikit-learn tree models. SHAP was initially developed by Shapley in 1953 and is based on the game theory [32]. It explains the prediction of a specific input (**X**) by calculating the impact of each feature on the prediction. The estimated Shapley values are calculated as follows (equation 9):



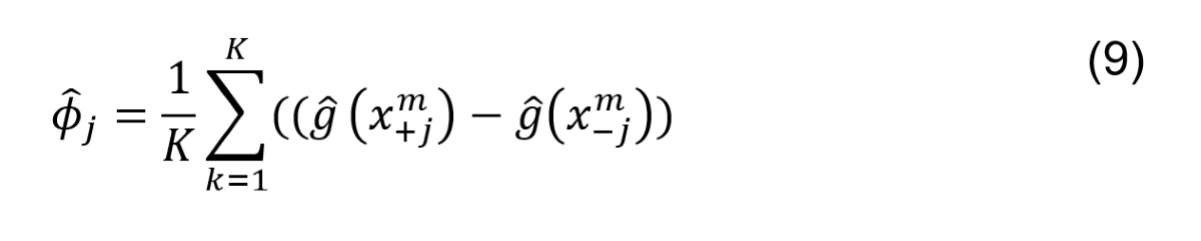



where 
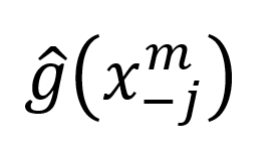
 is the prediction for *x*, but with a random number of feature values. TreeSHAP is used for gradient boosting models including XGBoost. It offers a rich visualization of each feature attribution and allows for partial dependence plots.

The TreeSHAP interaction values estimates as follows (equation 10):



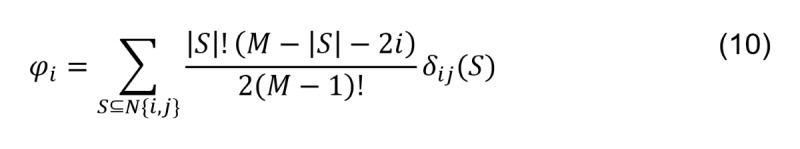



where *i* ≠ *j*, *δ_ij_*(*S*) = *f_x_*(*S* ∪ {*I*,*j*} – *f_x_*(*S* ∪ {*i*} – *f_x_*(*S* ∪ {*j*} + *f_x_*(*S*), *M* is the number of features, and *S* denotes all feature subsets. SHAP values advance the understanding of tree models by including feature importance, feature dependence plots, local explanations, and summary plots [30].

### Ethical Considerations

Ethics approval for the DEGS1 survey was obtained from the Charité – Universitätsmedizin Berlin Ethics Committee (EA2/047/08). All participants received written information and provided informed consent before the interview and examination. The analysis described here builds on a data set from the DEGS1 study, which was kindly provided by the Robert Koch Institute. This secondary analysis of anonymized data does not require a separate ethics vote.

## Results

### Characteristics of the DEGS1 Study Population

The mean age of the 5801 DEGS1 study participants was 44 years, with more than half of the population being female (n=3080, 53.1%). The mean stress level of the total population was 12.00 (95% CI 11.79-12.20): 11% (n=625) of the participants had “high chronic stress” (category 2), while 38% (n=2188) had “middle” (category 1), and 52% (n=2988) of them had “low chronic stress” (category 0). Most participants reported their general state of health as very good or good (79.3%, n=4599). Table 1 shows the weighted demographic, clinical, and laboratory characteristics of the participants.

### Results of the Machine Learning Analysis

**Figure 1:**
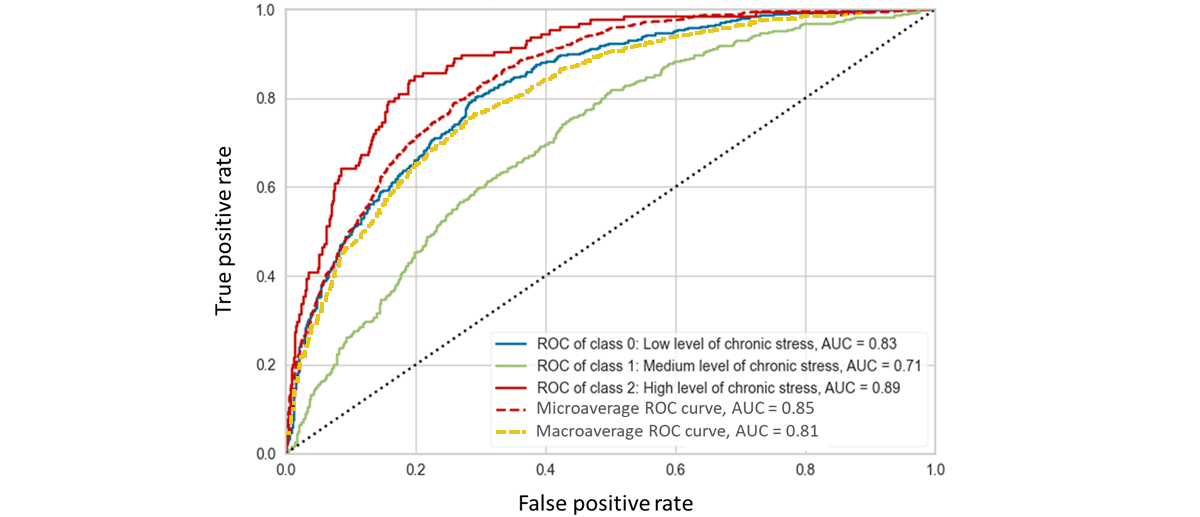
ROC curves for 3 classes using the XGBoost multiclass classifier. AUC: area under the receiver operating characteristic curve; ROC: receiver operating characteristic curve.

The evaluation metrics of the XGBoost model’s performance are presented in Table 3 differentiated by chronic stress classes. We see that the XGBoost model achieved the highest AUC score for class 2 with 0.89% and a good macroaverage AUC score of 81% for the overall model. The metrics for the 3 stress classes and the average results are reported in Table 3. The ROC curves for the multiclass chronic stress prediction of the XGBoost model are shown in Figure 1.

**Table 3:** Classification metrics: area under the receiver operating characteristic curve (AUC), precision, recall, specificity, and *F*_1_-score for XGBoost.

Measure	XGBoost			
	Class 0	Class 1	Class 2	Macroaverage
AUC	0.83	0.71	0.89	0.81
Precision	0.73	0.56	0.58	0.63
Recall	0.80	0.55	0.37	0.52
Specificity	0.90	0.38	0.26	0.78
*F*_1_-score	0.76	0.60	0.45	0.54

### Explanation of the Behavior of Individual Features

**Figure 2:**
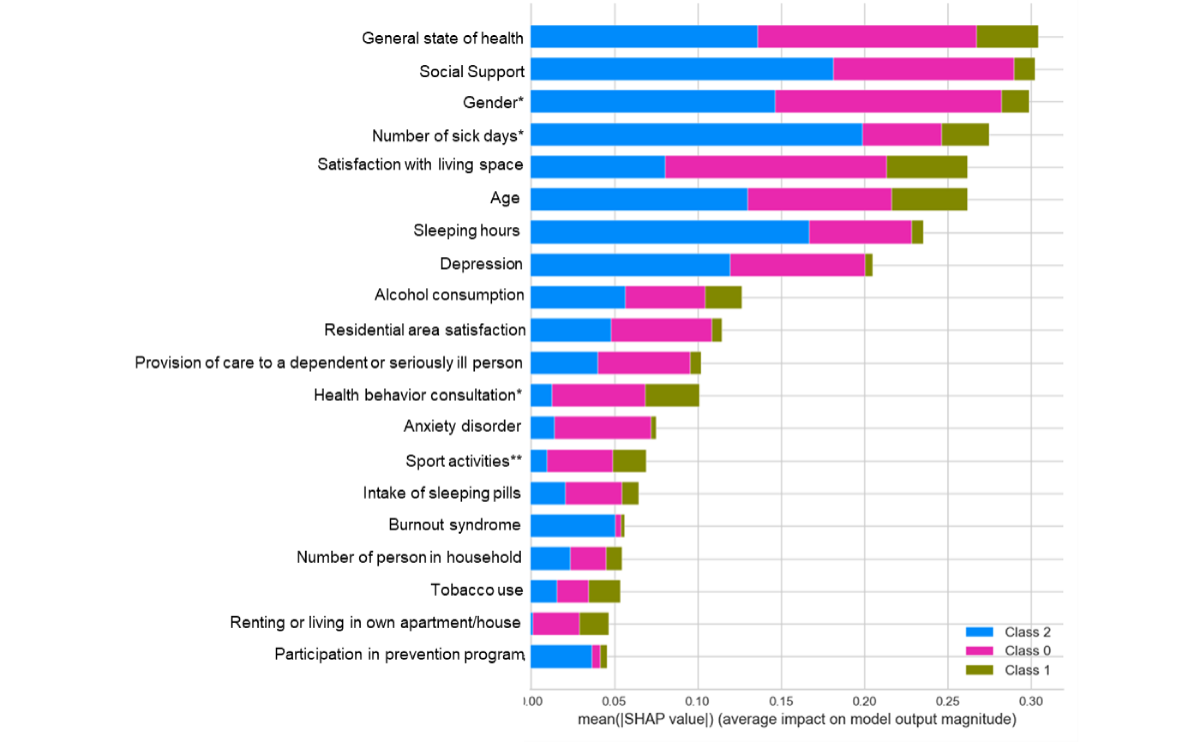
SHAP feature plot of the 20 most important features: relative importance of each feature based on the average absolute value of the SHAP values. SHAP: SHapley Additive exPlanations; XGBoost: Extreme Gradient Boosting. *In the past 12 months; **per week.

The result of the SHAP analysis is displayed in Figure 2. In this plot, the impact of a feature on the respective classes (stress classes 0-2) is stacked to illustrate the feature importance. This means that the features with large absolute Shapley values are more important than those with lower values. The plot shows that class 0 (low level of chronic stress) hardly uses the features gender, general state of health, satisfaction with living space, and social support. Class 2 as the high level of chronic stress uses the features number of sick days in the past 12 months, social support, sleeping hours per night in the past 4 weeks, gender, and general state of health. Interestingly, classes 0 and 2 use many identical features.

**Figure 3:**
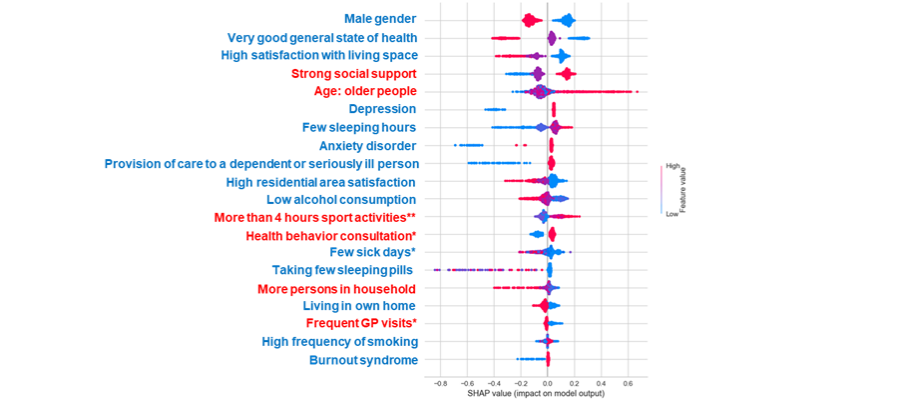
SHAP summary plot. Importance of the representative chronic stress features (top 20) in class 0: each dot is a Shapley value for a particular feature and reflects its impact on a specific class for a given instance, and dots stack up to show density. It is color-coded in accordance with the magnitude to which the value contributes to the model impact (red=high and blue=low). GP: general practitioner; SHAP: SHapley Additive exPlanations. *In the past 12 months; **per week.

**Figure 4:**
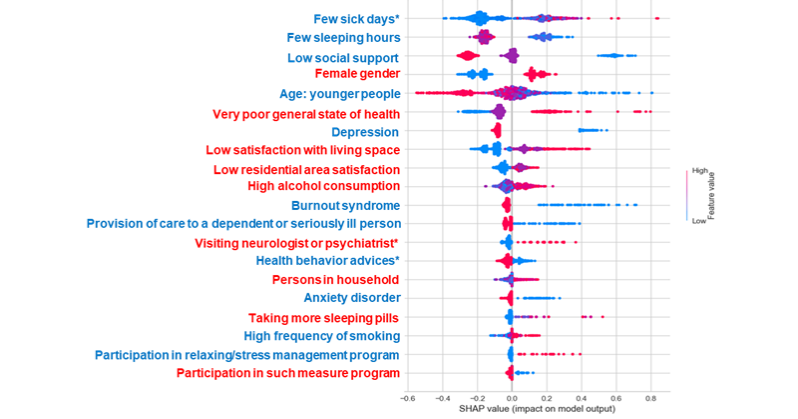
SHAP summary plot. Importance of the representative chronic stress features (top 20) in class 2: each dot is a Shapley value for a particular feature and reflects its impact on a specific class for a given instance, and dots stack up to show density. It is color-coded in accordance with the magnitude to which the value contributes to the model impact (red=high and blue=low). SHAP: SHapley Additive exPlanations. *In the past 12 months.

While the SHAP feature plot provides an overview of the role of each variable irrespective of the direction of these effects, the SHAP summary plot provides such additional information for classes. The impact distribution of each feature on the model output for classes with low and high levels of chronic stress is shown in Figures 3 and 4. Each row in this plot represents a single feature in order of their mean absolute SHAP values. It can be a negative or positive value and represents the importance of each feature. Each dot is a Shapley value for a particular feature and reflects its impact on a specific class for a given instance, and dots stack up to show density. It is color-coded in accordance with the magnitude to which the value contributes to the model impact (red=high and blue=low). The color is the actual feature value in the data set. For example, the red values for age as a continuous feature represent older people, while blue values represent younger people, and blue values for gender as a categorical feature (low value=1) represent males and red values (high value= 2) represent females. Overlapping points are jittered toward the y-axis, giving a sense of the distribution of the Shapley values per feature.

According to the SHAP summary plot result, gender is the most significant feature for class 0, and the number of sick days in the past 12 months has the highest impact on class 2. We note that the general state of health (shown in red) with high values has negative SHAP values and a relatively negative effect on the model for the low level of chronic stress and a positive impact (positive SHAP values) for class 2. Higher values on the social support scale have a positive impact on class 0 and negative effects on class 2, which means that chronic stress is less likely with strong social support.

## Discussion

### Principal Findings

To our knowledge, this is the first study to select the XGBoost algorithm as an ML multiclass classifier in the prediction of chronic stress as well as the SHAP method to interpret the model’s prediction. Based on nationally representative German data, chronic stress was predicted using 34 characteristics of adult participants. We identified male gender, a very good general state of health, high satisfaction with living space, strong social support, enough sleep, and more than 4 hours of sports activities per week as protective factors against chronic stress. These results are in line with those of other studies, which showed that resilience against chronic stress is promoted by social support, family connectedness, and friendship networks in the community [33-36]. For example, with a sample of 24,347 participants from the Canadian General Social Survey, Van der Horst et al [36] determined that good friendship networks are positively associated with less stress, better health, and more social support. A cross-sectional study of 538 nursing students from an Australian university showed that social support positively affect the psychological well-being [37].

Our ML approach allowed for the inclusion of a broad spectrum of individual characteristics, which comprised medical, lifestyle, living space, and social information, while other studies on chronic stress used multivariate models with fewer parameters only. For example, a large cross-sectional study with 34,129 participants from China, Ghana, India, Mexico, Russia, and South Africa showed positive associations of multimorbidity, stroke, depression, and hearing problems with perceived stress without assessing potential protective factors such as living space and social support [38]. A US cross-sectional telephone survey with 340,847 participants aged between 18 and 85 years documented that psychological well-being, especially stress, improved, but integrated only 5 parameters such as gender, employment status, partnership, and underage children in the household in their model analyzed [39]. In a study with 12,110 working adults from Minnesota, United States, a high level of perceived stress was associated with a higher-fat diet, less exercising, and being a smoker using a multivariate model with 6 variable topics but did not include medical and living circumstances [40].

### Strengths and Limitations

This study used the population-based, representative DEGS1 data set, which implies a low risk of selection bias; yet, the results may not be transferrable to other settings. The DEGS1 data, which were collected from 2008 to 2011, may not fully describe current living conditions in Germany, especially the potential effects of the pandemic, which were shown in other studies, were not measured [41]. In our study, the SHAP methodology allowed for a detailed visualization of single feature attributions, which improved the understanding of the ML model.

### Conclusions

In this study, we developed an XGBoost ML model to predict chronic stress in adults. The SHAP methodology identified various relevant factors protecting against chronic stress, which need to be considered when developing interventions for stress reduction and improving resilience.

## Appendix

Multimedia Appendix 1 Hyperparameter Tuning for XGBoost.

## Abbreviations

AUC: area under the receiver operating characteristic curve
DEGS1: German Health Interview and Examination Survey for Adults
GP: general practitioner
KNN: K-nearest neighbors
ML: machine learning
PrA: practice assistant
ROC: receiver operating characteristic
SHAP: SHapley Additive exPlanations
TICS-SSCS: Screening Scale of the Trier Inventory for the Assessment of Chronic Stress
XGBoost: Extreme Gradient Boosting
