# Supplementary material for: Prediction of Chronic Stress and Protective Factors in Adults: Development of an Interpretable Prediction Model Based on XGBoost and SHAP Using National Cross-sectional DEGS1 Data
Source: JMIR AI. 2023 May 16;2:e41868. doi: 10.2196/41868 (PMC11041452; doi:10.2196/41868)
Supplement: Multimedia Appendix 1 [file ai_v2i1e41868_app1.pdf]

Appendix 1: Hyperparameter Tuning

The hyperparameter learning rate needs to be tuned to prevent the model from quickly fitting and then overfitting the training dataset. In this study, we used the grid search method, which is a type of brute-force technique. Typically, it is used to search through a manually defined subset of hyperparameters of a learning algorithm [1].

We set up a parameter grid to search optimal parameters as follows:

```
# A parameter grid for XGBoost
PARAMETERS = {

             'subsample' : [0.6, 0.8, 1.0],
             'colsample_bytree' : [0.6, 0.7, 1.0],
             'max_depth' : [3,4,5],
             'min_child_weight' : [1.0, 5.0, 10.0],
             'learning_rate' : [0.01, 0.1, 0,3],
             'reg_lambda' : [1.0, 2.0, 5.0, 10.0],
             'n_estimators' : [100, 500, 1000]
             }
```

After setting up the XGboost classifier using sklearn's API of XGBoost, we used the stratified K- fold with K=5 in order to get more reliable result and the AUC as a scoring function. Scikit-learn’s GridSearchCV function is used to find the best hyperparameter value combination from a predefined parameter for each model [2].

The results showed the best estimator and best hyperparameters as follows:

Best estimator:

```
XGBClassifier(base_score=0.5, booster='gbtree', colsample_bylevel=1,
       colsample_bytree=0.7, gamma=0, learning_rate=0.03, max_delta_step=0,
       max_depth=5, min_child_weight=3, missing=None, n_estimators=1000,
       n_jobs=1, nthread=1, objective='multi:softmax', random_state=0,
       reg_alpha=0, reg_lambda=2, scale_pos_weight=1, seed=None,
       silent=True, subsample=0.8)
```

Best hyperparameters:

```
{'learning_rate: 0.3', 'min_child_weight': 3, 'subsample': 0.8, 'reg_lambda': 2
, 'max_depth': 5, 'colsample_bytree': 0.7, 'n_estimators': 1000}
```

References

1. I. GPB. Hyperparameter Optimization in XG Boost for Insurance Claim Prediction. JARDCS. 2020; 12:1510–7. doi: 10.5373/JARDCS/V12SP4/20201630.
2. scikit-learn. sklearn.model_selection.GridSearchCV [updated 10 Feb 2023; cited 13 Feb 2023]. Available from: https://scikit-learn.org/stable/modules/generated/sklearn.model_selection.GridSearchCV.html.
